# Degradation Study of Thin-Film Silicon Structures in a Cell Culture Medium

**DOI:** 10.3390/s22030802

**Published:** 2022-01-21

**Authors:** Huachun Wang, Jingjing Tian, Bingwei Lu, Yang Xie, Pengcheng Sun, Lan Yin, Yuguang Wang, Xing Sheng

**Affiliations:** 1 Department of Electronic Engineering, Beijing National Research Center for Information Science and Technology, Center for Flexible Electronics Technology, IDG/McGovern Institute for Brain Research, Tsinghua University, Beijing 100084, China; whc17@mails.tsinghua.edu.cn (H.W.); yangxie2019@163.com (Y.X.); 2 Department of Medical Science Research Center, Peking Union Medical College Hospital, Chinese Academy of Medical Sciences & Peking Union Medical College, Beijing 100730, China; tianjing311@163.com; 3 Center for Mechanics and Materials, Department of Engineering Mechanics, Tsinghua University, Beijing 100084, China; thulubw@foxmail.com; 4 The Key Laboratory of Advanced Materials of Ministry of Education, State Key Laboratory of New Ceramics and Fine Processing, School of Materials Science and Engineering, Tsinghua University, Beijing 100084, China; spc18@mails.tsinghua.edu.cn (P.S.); lanyin@tsinghua.edu.cn (L.Y.); 5 National Engineering Laboratory for Digital and Material Technology of Stomatology, Peking University School and Hospital of Stomatology, Beijing 100082, China; wangyuguang@bjmu.edu.cn

**Keywords:** thin-film silicon, cell culture medium, biodegradation, transient electronics, biocompatibility

## Abstract

Thin-film silicon (Si)-based transient electronics represents an emerging technology that enables spontaneous dissolution, absorption and, finally, physical disappearance in a controlled manner under physiological conditions, and has attracted increasing attention in pertinent clinical applications such as biomedical implants for on-body sensing, disease diagnostics, and therapeutics. The degradation behavior of thin-film Si materials and devices is critically dependent on the device structure as well as the environment. In this work, we experimentally investigated the dissolution of planar Si thin films and micropatterned Si pillar arrays in a cell culture medium, and systematically analyzed the evolution of their topographical, physical, and chemical properties during the hydrolysis. We discovered that the cell culture medium significantly accelerates the degradation process, and Si pillar arrays present more prominent degradation effects by creating rougher surfaces, complicating surface states, and decreasing the electrochemical impedance. Additionally, the dissolution process leads to greatly reduced mechanical strength. Finally, in vitro cell culture studies demonstrate desirable biocompatibility of corroded Si pillars. The results provide a guideline for the use of thin-film Si materials and devices as transient implants in biomedicine.

## 1. Introduction

Silicon (Si)-based materials and devices such as electrodes, diodes, transistors, and circuits have a broad range of applications in biomedical research, owing to their diverse spectrum of physical and chemical properties, mature manufacturing, and favorable biocompatibility [1]. In addition, micropatterned 1D, 2D, and 3D Si structures are attractive, label-free, and scalable sensing platforms, owing to their distinctive optical and/or electrical properties and high surface-to-volume ratio, which make them broadly interesting for biomedical applications in healthcare scenarios [2,3,4]. For example, Si pillars or nanowire field-effect transistors have been promisingly developed as potentiometric sensor devices for detecting a copious number of chemical and biological species, such as ions, DNA, proteins, and antibodies/antigens [5,6,7]. Other remarkable examples include the development of neural modulators [8,9], scaffolds [10,11,12], and implantable energy harvesters [13,14]. More recently, thin-film Si devices have been discovered to present notable degradation behavior in biological environments, giving birth to a new field known as “transient electronics” [15,16,17]. Unlike conventional electronics, which pursues chronic operation and avoids any change or degradation in either material constituents or device performance, physically transient electronic systems based on thin-film Si are designed to perform in an opposite manner, with devices completely or selectively disappearing over a well-defined time frame in a physiological environment (pH 7.2–7.4) through a hydrolysis process. According to the proposed hydrolysis reaction (Si + 4H_2_O → Si(OH)_4_ + 2H_2_), the final dissolution product—silicic acid (Si(OH)_4_)—is biocompatible, resorbable, and naturally present in the human body [15,18]. Integrated with biodegradable electrodes, dielectrics, and substrates [19,20,21], such thin-film Si-based transient electronic devices can be used as diagnostic or therapeutic implants, effectively preventing secondary surgical intervention and greatly minimizing the health risks caused by infections [22,23].

Understanding the hydrolytic behavior of Si-based materials and devices is crucially important for the rational design of transient implants with desirable degradation processes. In fact, the hydrolytic kinetic process is highly complicated and is closely associated with numerous factors with nano-, micro-, meso-, and even macro-scale effects. Previous studies have revealed that chemical species, ion concentrations, pH levels, doping types, dopant concentrations, and pattern geometries can all remarkably affect the dissolution behavior of Si in aqueous solutions. In general, anions with higher concentrations—such as chlorides and phosphates—as well as elevated temperatures and pH levels, can increase hydrolysis rates [24]. On the other hand, higher dopant (e.g., phosphorus for n-type doping, boron for p-type doping) concentrations could dramatically decrease dissolution rates—for example, from ~2.8 nm/day for lightly boron-doped (~10^17^ cm^−3^) Si to ~0.16 nm/day for heavily boron-doped (~10^20^ cm^−3^) Si [25]. Another recent study reports an interesting finding that micropatterned p-type Si films with smaller areas present lower dissolution rates, and the introduction of stirring further slows down the degradation [26]. Previous in vitro studies mostly examined the dissolution of planar Si membranes in phosphate-buffered saline (PBS) solution for a relatively short period (<14 days); few studies have focused on their long-term degradation behavior in biologically relevant environments such as cell culture media (e.g., Dulbecco’s modified Eagle medium, or DMEM). In most cases, Si membranes exposed to DMEM are only used for the demonstration of biocompatibility, or for the measurement of dissolution rate [18,27]. Additionally, evolutions of other physical and/or chemical properties, such as mechanical properties and compositional states—especially for Si microstructures, the degradation behaviors of which will greatly affect their sensing stability and repeatability—have received little attention in the past.

In this study, we experimentally explored the long-term (within 21–30 days) degradation behavior of planar Si thin films as well as structured Si pillar arrays in a cell culture medium (Dulbecco’s modified Eagle medium (DMEM)). The dissolution of Si in DMEM led to dramatic transformations in surface morphology, physical and chemical states, mechanical strength, and electrochemical impedance. In addition, we found that Si pillar arrays experience a more severe degradation than planar Si films. This study establishes a basic understanding of degradation for Si microstructures, and offers instructions on the design of advanced transient implants for versatile applications.

## 2. Materials and Methods

### 2.1. Fabrication of Planar Si Thin Films and Si Pillar Arrays, and Their Degradation Experiments

The fabrication of Si thin-film structures starts with silicon-on-insulator (SOI) wafers (p-type, boron doping concentration ~10^15^ cm^−3^, resistivity 1–10 Ω·cm, (100) orientation, Soitec, France). The thicknesses of the top Si device layers are 2 µm and 5 µm for planar Si films and Si pillar arrays, respectively. The top Si layers are patterned by photolithography (photoresist SPR220-v3.0, 3–6 µm, Rohm & Haas, Philadelphia, PA, USA) and reactive-ion etching (SF_6_ plasma, 150 sccm, 80 mTorr, 100 W, etching rate ~1 µm/min). For planar Si films, the top Si layers are patterned into squares with different sizes (100 × 100 µm^2^ and 1000 × 1000 µm^2^). For Si pillar arrays, the top Si layers (5 µm) are patterned into arrays of pillars with 2 µm in height, 0.5 µm in diameter, and a period of 7 µm. The total surface area of the fabricated Si sample is 1.5 × 1.5 cm^2^.

### 2.2. In Vitro Degradation of Thin-Film Si Structures

Patterned thin-film Si structures were immersed in a phosphate-buffered saline solution (PBS solution, 0.1 M, pH 7.2–7.4, including 136 mM NaCl, 10 mM Na_2_HPO_4_/NaH_2_PO_4_, and 2.6 mM KCl, Solarbio, Beijing, China) or a typical cell culture medium—the high-glucose Dulbecco’s modified Eagle medium (DMEM; L-Glutamine, 4.5 g/L D-glucose, 0.11 g/L sodium pyruvate, supplemented with 10% fetal bovine serum and 1% penicillin–streptomycin, from Thermo Fisher Scientific company, Waltham, MA, USA)—for degradation tests. Samples were kept in an incubator (95% humidity, 5% CO_2_, 37 °C). Solutions were replaced every two days to maintain constant concentration during the test. All of the reported statistical data were measured an average of at least 3 times, with error bars representing the standard deviation.

### 2.3. Characterizations of Materials

The thickness profiles of the Si thin films were measured by an Alpha-Step profilometer (stylus force: 0.1 mg, scan speed: 0.03 mm/sec). Scanning electron microscopy (SEM) and energy-dispersive X-ray spectroscopy (EDS) images were collected using a Zeiss Merlin Compact and a Gemini SEM 500 field-emission scanning electron microscope after five minutes (5~10 nm in thickness) of magnetron sputtering of platinum for all samples. X-ray photoelectron spectroscopy (XPS) was conducted using a Thermo Scientific ESCALAB 250XI (UK) equipped with an Al Kα source gun, and the data were processed with peak-fitting software (XPSPEAK 4.1). Atomic force microscopy (AFM, SPM Contacting model) and Kelvin probe force microscopy (KPFM, SPM Tapping Mode) were conducted using a Bruker Dimension Icon, and the data were analyzed using NanoScope Analysis software.

### 2.4. Mechanical Characterization

Mechanical compression tests were performed via nanoindentation at room temperature. Planar Si films were measured using an XP CSM (continuous stiffness measurement) interactive nanoindenter equipped with a sharp AccuTip (Keysight Technologies G200), under an average down speed of 2 nm/s. Si pillar samples were tested using an SEM (Quanta FEG450) equipped with a Hysitron PicoIndenter 85. A flat-ended diamond conical punch with a diameter of 5 µm (larger than the tested Si pillar) and the compressed pillars were observed in situ via the SEM operated at 5 kV. Through the visual control of the SEM, the punch was accurately positioned over the pillar, and then the compression tests were conducted under a constant strain rate (average down speed: 1~2 nm/s), and with a maximum load force of 8 mN.

### 2.5. Measurement of Electrochemical Impedance Spectra (EIS)

The EIS tests for the probe-shaped Si electrodes were performed using a Gamry Interface 1000E Potentiostat (in the open-circuit potential model) in PBS solutions at room temperature via a three-electrode configuration, with the Si probe serving as the working electrode, a standard Ag/AgCl electrode as the reference electrode, and a Pt sheet electrode as the counter-electrode. The EIS results were obtained by varying the frequency over a range from 10 Hz to 10 kHz.

### 2.6. Cell Culture

Si pillar samples were first immersed in a dopamine solution (2 mg/mL in 10 mM Tris-HCl buffer solution, pH 8.5) for 20 min, resulting in a hydrophilic surface coated with polydopamine molecules for improved cell adhesion [28]. Then, the samples were sterilized in 75% ethanol for 30 min followed by ultraviolet (UV) irradiation for another 30 min. Finally, the human-bone-marrow-derived mesenchymal stem cells (hBMSCs) (#7500, ScienCell, Carlsbad, CA, USA) were directly inoculated on silicon pillar array samples in DMEM in a CO_2_ incubator. The cell viability was evaluated after culturing for 7, 14, and 21 days. The fluorescent live/dead assay kit, calcein-AM, and propidium iodide (KeyGEN BioTECH, Nanjing, China) PBS solutions (1:200 *v*/*v* dilution) were used to stain viable (fluoresce bright green) and dead (fluoresce bright red) cells, respectively. A fluorescence microscope (Olympus Corporation, Tokyo, Japan) was used to detect the distribution of living and dead cells.

## 3. Results and Discussion

### 3.1. Characterization of Surface Topography and Dissolution Rate

**Figure 1:**
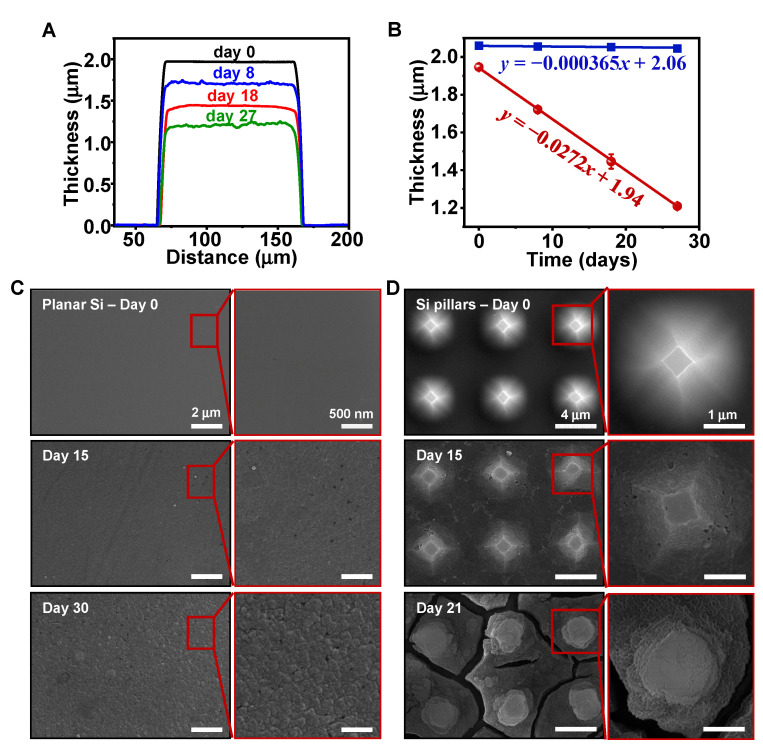
(**A**) Line scan profiles for planar Si immersed in DMEM at different stages. (**B**) Measured changes in thickness as a function of immersion time for planar Si samples in DMEM (red) and PBS (blue) solutions. (**C**,**D**) Top-view SEM images of surfaces for (**C**) planar Si and (**D**) Si pillars immersed in DMEM at different stages.

In previous works, most in vitro studies have been mainly focused on the hydrolysis behavior of planar Si patterns in aqueous solutions such as PBS, which only contains small varieties of inorganic cations and anions [29]. Here we primarily investigated the degradation of Si structures in DMEM solution (pH 7.2–7.4, at 37 °C), which is a standard medium for cell culture and is much more biologically relevant. Compared to PBS, the DMEM comprises much more complex compositions, including proteins, amino acids, growth factors, glucose, vitamins, etc., bearing a close resemblance to actual physiological environments. As shown in Figure 1A, the line scan profiles measured by a profilometer provide a set of changes in thickness for planar Si films at different stages of immersion in DMEM. Figure 1B compares measured dissolution rates for planar Si in PBS (blue) and DMEM (red). The solid lines were obtained by fitting a linear function, and the slopes demonstrate quite stable dissolution rates of planar Si, which are 0.365 nm/day in PBS and 27.2 nm/day in DMEM at 37 °C. Markedly, the DMEM solution greatly facilitates the dissolution of Si—by almost 75-fold compared to the PBS solution. This difference in the degradation rate can be ascribed to the complicated and diverse components in DMEM, which weaken the interior bonds of Si atoms and, thus, promote the reaction to form silicic acid (Si(OH)_4_) [15]. Quantifying the degradation rate for Si samples with pillar structures is also critically important, but the direct characterization of thickness is difficult because of the rough surface. Possible means of characterization will be explored in future works.

The SEM images in Figure 1C,D illustrate the microscale structural evolution during the degradation process in DMEM for planar Si films and Si pillar structures, respectively. For planar Si in Figure 1A, the as-prepared surface (day 0) is flat and smooth, and then the surface roughens and becomes uneven after immersion for 15 days. After 30 days, granular-like microstructures with cracks indicate that prominent corrosion occurs on the Si surface during the degradation. On the other hand, morphological changes in Si pillar arrays are investigated in Figure 1B. Compared to the planar Si, Si pillar arrays are much more severely corroded (Figure 1B). In particular, after immersion in DMEM for 21 days, cracks and gaps develop around the pillars, and peculiar needle-like structures appear all over the surface.

**Figure 2:**
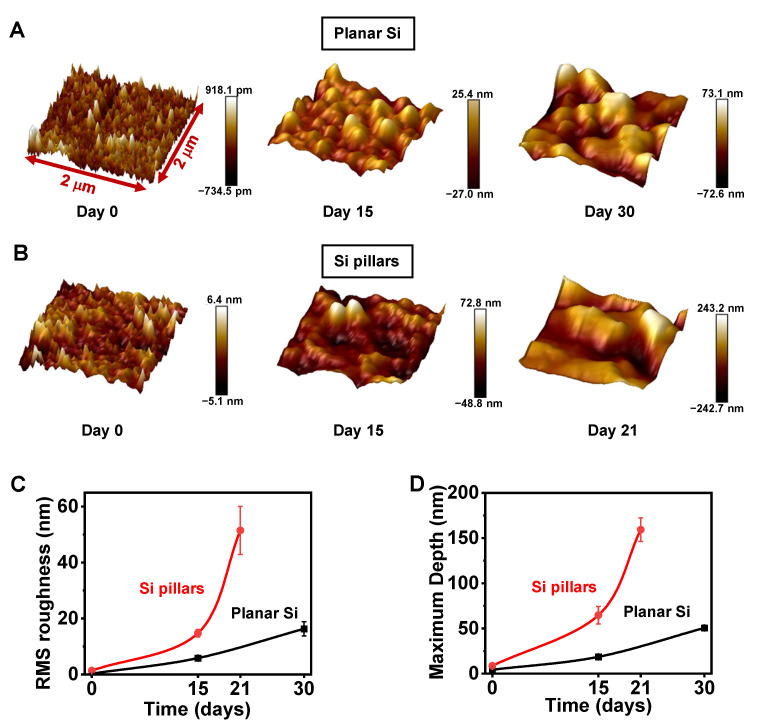
(**A**,**B**) Three-dimensional AFM images showing the surface morphologies of (**A**) planar Si and (**B**) Si pillars during degradation in DMEM. (**C**,**D**) Summarized (**C**) RMS roughness and (**D**) profile depth based on AFM images.

To quantify these structural changes, AFM scanning was utilized to reveal the morphological characteristics by imaging the sample surface at the sub-micrometer level. The scanned surface topography (area 2 × 2 µm^2^) before and after degradation in DMEM is shown in Figure 2A,B. The surfaces of both planar Si and Si pillar arrays (in the region between the pillars) demonstrate increased root-mean-square (RMS) roughness, consistent with the SEM results in Figure 1C,D. Clearly, Si pillar samples encounter stronger corrosion and exhibit greater RMS roughness (50.17 nm for Si pillars on day 21, compared to 16.04 nm for planar Si on day 30), as illustrated in Figure 2C. Additionally, depth analyses summarized in Figure 2D show that the pattern height of Si pillar arrays is much larger than that of planar Si films. Collectively, Si microstructures with pillars exhibit a faster and more severe degradation in DMEM than the planar Si films; this is probably attributable to the large surface area of the Si pillar samples, as well as the nanoscale damage to the Si surface during dry etching when preparing the pillars.

### 3.2. Analysis of Surface Chemistry and Physical States

**Figure 3:**
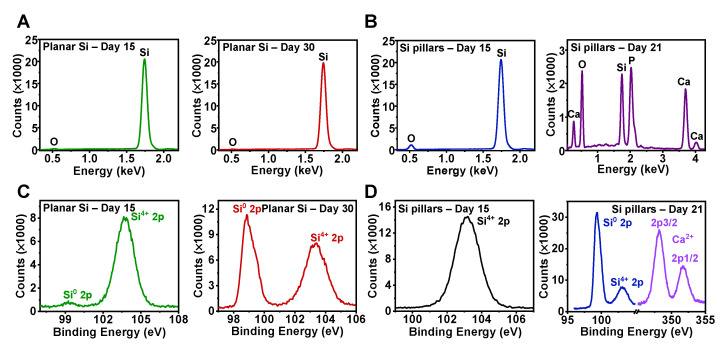
Characterization of surface chemistry associated with degradation in DMEM for different Si samples. (**A**,**B**) EDS data for (**A**) planar Si and (**B**) Si pillars. (**C**,**D**) XPS data for (**C**) planar Si and (**D**) Si pillars.

We performed EDS and XPS analysis to further exploit the chemical variations in Si surfaces during the degradation (Figure 3). Figure 3A,C present EDS and XPS results for planar Si films after exposure to DMEM for 15 (left) and 30 (right) days, respectively. The representative oxygen (O) and Si^4+^ peaks suggest that oxidation reactions occurred on the Si surface during the degradation. As regards the Si pillar arrays (Figure 3B,D), on the one hand, higher O peak intensity in the EDS spectra (Figure 3B, left) and larger area under the Si^4+^ in the XPS spectra (Figure 3D, left) data indicate a higher degree of oxidation. On the other hand, the EDS profiling (Figure 3B, right) shows the presence of additional foreign phosphorus (P) and calcium (Ca) peaks on the Si pillar arrays surface after degradation for 21 days, which is partially consistent with the XPS spectra (Figure 3D, right), with strong typical calcium salt (CaCO_3_, Ca(OH)_2_, etc.)-related peaks (Ca^2+^, 2p1/2 351.2 eV, 2p3/2 348.2 eV). In agreement with previous studies [24], metal and phosphate ions (Ca^2+^, PO_4_^3−^, etc.) in DMEM accumulate on the Si surface and promote the Si degradation process. As inferred by the above chemical component analysis, other inorganic salts (e.g., CaSO_4_, NaH_2_PO_4_, NaHCO_3_) adsorbed onto Si surfaces as biochemical residues may also exist from the basic ingredients of DMEM solution, such as L-glutamine and sodium pyruvate, but those corresponding signals may be too weak to be detected. These findings highlight the complex mixture of oxides and salt corrosion products generated on Si microstructures during their degradation in DMEM.

**Figure 4:**
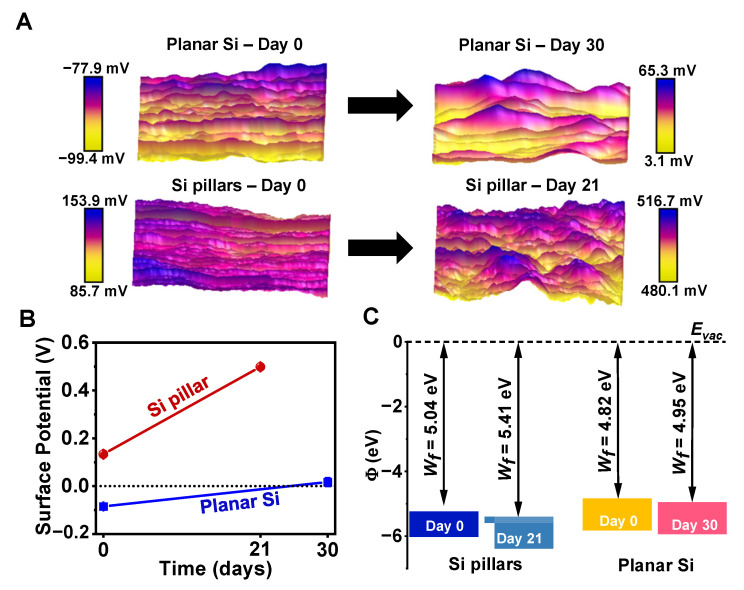
(**A**) KPFM images showing surface potential maps (area 2 µm × 2 µm) for planar Si (**top**) and Si pillars (**bottom**) before (**left**) and after (**right**) degradation in DMEM. (**B**) Values of surface potentials at different stages during degradation. (**C**) Work functions of planar Si and Si pillars before and after degradation in DMEM, obtained from the KPFM results. The probe is calibrated with a standard Au sample, with Wf = 5.2 eV.

It is known that semiconductor surface states or surface potentials play an important role in Si electronics. The electronic and optical properties of semiconductor homojunctions and heterojunctions can be controlled by altering the energy band alignment and carrier distributions at the junction interface [30]. In physiological environments, Si-based implantable devices may also establish functional interfaces with biological molecules, cells, tissues, and organs, and offer localized biophysical cues to the biosystems [1]. Therefore, understanding surface electronic states is crucial for Si-based transient implants at the biointerfaces. For instance, the energy band positions relative to the solution redox potentials determine the electrochemical or photoelectrochemical reactions at the Si–solution interface. KPFM is a powerful tool to measure the semiconductor work function (Wf; energy difference between the Fermi energy and the vacuum level) by recording the contact potential difference (CPD) between the probe and the sample surface [31]. In order to investigate the changes in surface states, we performed KPFM on the surfaces of Si samples (scanning area 2 × 2 µm^2^) before and after degradation in DMEM. Figure 4A maps the CPD distributions (calibrated with a standard Au film sample with Wf = 5.2 eV), and all of the samples yielded growing surface potentials (with mean values extracted from Figure 4A), with ΔV = 101.7 mV for the planar Si film and ΔV = 365.1 mV for the Si pillar arrays (Figure 4B). The corresponding work functions are calculated and summarized in Figure 4C. In this comparative study, one can see that the work functions for both Si samples slightly increased over the course of degradation process. The work function of the corroded planar Si sample (at day 30) remained larger than that of the original Si pillar sample (at day 0); this result is probably attributable to the surface contamination and/or oxidation occurring during the dissolution process. It is also known that the doping condition affects the state of the Si as well as its degradation rate [25,26]. Combining the surface chemical and physical analysis, our results indicate that the degradation causes dramatic changes of surface states and alters the energy band or electrical properties at the biointerface, and will eventually affect the operational performance of the implantable Si devices.

### 3.3. Mechanical and Electrochemical Properties

**Figure 5:**
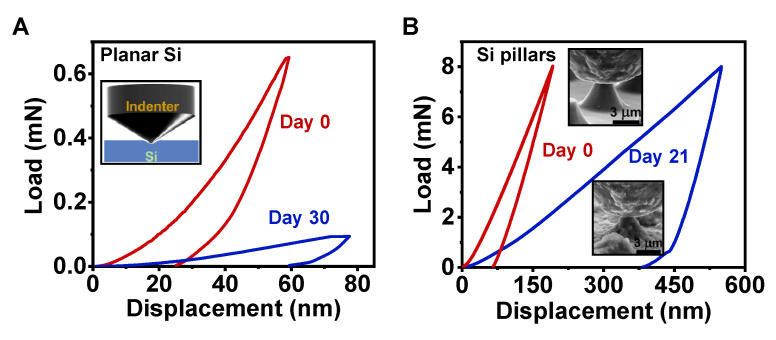
Experimentally measured load–displacement curves for (**A**) planar Si and (**B**) Si pillars before (red) and after (blue) degradation in DMEM. Inset in (**A**): scheme for test setup. Insets in (**B**): SEM images showing the indenter on Si pillars.

Changes in microstructural and chemical composition also induce degradation of the mechanical properties of Si-based transient devices, which have rarely been explored previously. For example, Si-based implants have been extensively developed for use as chronic intracortical electrodes (e.g., silicon micromachined Utah arrays) to decode neural signals [32], as well as bio-microelectromechanical systems (Bio-MEMS) for biosensing [33]. In these scenarios not only chemical corrosion, but also mechanical failure, greatly challenges the reliability, stability, and longevity of devices. As shown in Figure 5, we performed nanoindentation (known as a depth-sensing indentation system) to explore the mechanical properties of Si samples at a micro/nano scale. For planar Si films, microcompressions are imposed with a sharp AccuTip loaded in a continuous stiffness measurement interactive mode. Figure 5A plots load–displacement curves during a representative cycle. The indentation depth for planar Si was ~59 nm under the maximum load of 650 µN. After degradation for 30 days, there was a larger indentation depth (78 nm) under a much lower load (94 µN). Tests were repeated at least three times at various positions for each sample, and the obtained Young’s modulus and hardness of degraded planar Si were 21.6 ± 5.1 GPa and 1.12 ± 0.11 GPa, respectively. These mechanical properties are almost one order of magnitude lower than those of as-prepared planar Si films (modulus 156.9 ± 2.4 GPa and hardness 14.75 ± 0.54 GPa). For Si pillar arrays, we performed mechanical tests using a flat-ended diamond conical punch with a diameter of 5 µm inside an in-s u SEM visual system, so that the punch could be accurately positioned over a pillar; the load–displacement curves are given in Figure 5B. When reaching the same maximum load of 8 mN, the Si pillars after degradation (for 21 days) presented a much larger displacement (550 nm) than the original structures (192 nm), indicating that Si pillars undergo a significant decrease in compressive strength during degradation. These studies on the mechanical behaviors of both planar Si and Si pillars during biodegradation provide insights into the design of Si implants that are mechanically compliant with biosystems.

**Figure 6:**
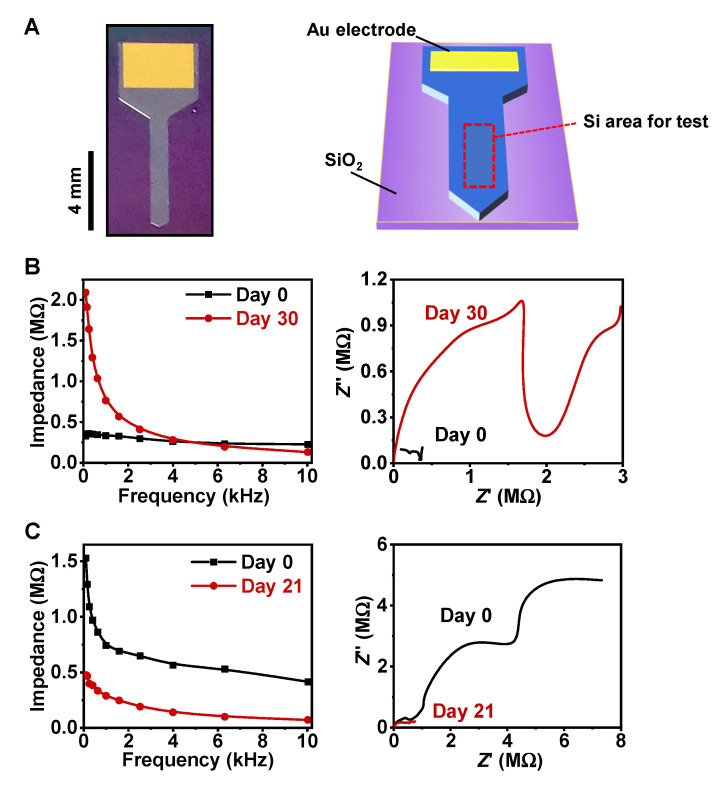
(**A**) Optical image (left) and schematic diagram (right) of a Si-based probe prepared for electrochemical tests. (**B**,**C**) Measured impedance for (**B**) planar Si and (**C**) Si pillars before (black line) and after degradation (red line) in DMEM.

The chemical and structural evolutions of these Si samples also induce changes in electrochemical properties at the Si–solution interface [34]. We prepared needle-shaped Si probes similar to Michigan electrodes, as shown in Figure 6A. The front area of the Si probes was designed and fabricated to form planar or pillar arrays. Immersed in PBS at room temperature, the electrochemical impedance was measured with a three-electrode configuration. Figure 6B,C present the measured impedance results (left: Bode spectra; right: Nyquist spectra) for planar Si and Si pillars, respectively. The charge-transfer resistance can be estimated from the semicircle region of the Nyquist plots, which is related to the electrochemical activity at the Si–solution interfaces. Based on the Nyquist spectra, after 30-day immersion in DMEM, the planar Si sample achieved higher charge-transfer resistance, indicating lower conductivity with poor transport of charge carriers. In contrast, the Si pillar sample obtained lower charge-transfer resistance after 21-day immersion, showing excellent conductivity with faster transfer of charge carriers. In short, after degradation in DMEM, the planar Si sample exhibited a substantial increase in impedance, while the Si pillar sample showed dramatically reduced impedance. This striking difference in the change in impedance is probably associated with the micro/nanostructural evolution of the different samples. For planar Si, the enhanced impedance may be caused by thin insulating oxides formed on the surface. In contrast, the degradation of Si pillars causes notable crack formation (Figure 1D), which could significantly increase the surface area and decrease the interface impedance.

### 3.4. In Vitro Biocompatibility Test

**Figure 7:**
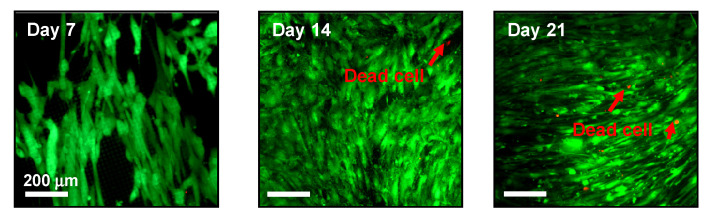
In vitro biocompatibility tests for Si pillar samples, showing fluorescent images of hBMSCs in live (green fluorescence)/dead (red fluorescence) viability assay at different times (day 7, day 14, and day 21).

Considering the remarkable transformation of the surface topography and chemical and physical states during the degradation process, it is critical to evaluate the biocompatibility of Si pillar structures. Here, the common live/dead assay was carried out to measure cell viability and assess cytotoxicity in vitro. hBMSCs were cultured in DMEM, with fluorescent images taken on days 7, 14, and 21, as shown in Figure 7. Calcein-AM (Ex/Em, 485 nm/535 nm) and propidium iodide (Ex/Em, 530 nm/620 nm) were applied to stain live (green) and dead (red) cells, respectively. Overall, hBMSCs were successfully cultured, and proliferated significantly from 7 to 21 days in DMEM, in spite of severe degradation occurring on the surface of these Si pillar arrays. The reported biocompatibility of the Si films was consistent with the literature [23]. Although the number of dead cells (red staining) increased due to the natural apoptosis during the long-term culturing, the ratio of dead cells to live cells was still very low. In terms of the cellular morphology, cell quantity, and viability, there were no obvious toxic effects throughout the whole experimental duration, confirming that the degradation reactions that occurred at the Si–solution interface are fully biocompatible and harmless to cell metabolism.

## 4. Conclusions

In summary, this work systematically studied the degradation behavior of micro- structured Si films in a standard cell culture environment, which is more pertinent to in vivo biomedical applications. It was discovered that these crystalline Si samples exhibit a much larger degradation rate in DMEM than in PBS. For both planar Si and Si pillar samples, cracks and irregular microstructures with high surface roughness were generated on the surface, and samples with Si pillars underwent more significant degradation due to their microstructure. By virtue of chemical analyses, the introduced foreign elements—including O, Ca, and P—suggest that corrosion products with complex mixtures of oxides or salt compounds formed after contacting the physiological solution. These dramatic morphological and chemical evolutions modified the physical states of the Si surface by increasing the work function. Furthermore, the cracks and corroded surfaces also caused great reduction in mechanical strength. Naturally, all of the above highlighted physical or chemical changes can produce huge impacts on the operational performance of microstructured Si film devices—especially for sensing devices whose working mechanism strongly depends on superficial structures and surface states. From the perspective of our results, a comprehensive and systematic performance evaluation technology is therefore required for sensor devices used in biological conditions. Finally, in vitro cell culture studies provide evidence of the desirable biocompatibility of Si pillar arrays even after severe surface degradation. These collective investigations have relevance to the further development of Si-based transient implantable electronics used in broad biomedical applications.
